# Role of Glutathionylation in Infection and Inflammation

**DOI:** 10.3390/nu11081952

**Published:** 2019-08-20

**Authors:** Paola Checconi, Dolores Limongi, Sara Baldelli, Maria Rosa Ciriolo, Lucia Nencioni, Anna Teresa Palamara

**Affiliations:** 1 IRCCS San Raffaele Pisana, Department of Human Sciences and Promotion of the Quality of Life, San Raffaele Roma Open University, 00166 Rome, Italy; 2 Department of Biology, University of Rome Tor Vergata, 00133 Rome, Italy; 3 Department of Public Health and Infectious Diseases, laboratory affiliated to Istituto Pasteur Italia-Fondazione Cenci Bolognetti, Sapienza University of Rome, 00185 Rome, Italy

**Keywords:** glutathione, glutathionylation, redox signaling, infection, inflammation

## Abstract

Glutathionylation, that is, the formation of mixed disulfides between protein cysteines and glutathione (GSH) cysteines, is a reversible post-translational modification catalyzed by different cellular oxidoreductases, by which the redox state of the cell modulates protein function. So far, most studies on the identification of glutathionylated proteins have focused on cellular proteins, including proteins involved in host response to infection, but there is a growing number of reports showing that microbial proteins also undergo glutathionylation, with modification of their characteristics and functions. In the present review, we highlight the signaling role of GSH through glutathionylation, particularly focusing on microbial (viral and bacterial) glutathionylated proteins (GSSPs) and host GSSPs involved in the immune/inflammatory response to infection; moreover, we discuss the biological role of the process in microbial infections and related host responses.

## 1. Introduction

### Glutathione, Cellular Thiols, and Glutathionylation

The tripeptide γ-l-glutamyl-l-cysteinyl-glycine, or glutathione (GSH), is the most abundant eukaryotic intracellular thiol, found in a concentration range of 1–10 mM. It is mainly localized in the cytosol (up to 90%), but it is also present in the mitochondria (10%), the endoplasmic reticulum, and the nucleus in a small percentage [1]. The antioxidant activity of GSH is probably the best known and popular function of this molecule; in fact, thanks to the reactivity and the reducing ability of its cysteinyl residue’s sulfhydryl (-SH) group, it can quickly react with and neutralize potentially dangerous molecules, both of endogenous and exogenous origin, as reactive oxygen and nitrogen species (ROS and RNS, respectively), xenobiotics, and metals [2].

However, today it is well known that the essential role of GSH overcomes its scavenger activity and it plays a crucial role in cell fate, regulating processes such as proliferation, differentiation, and apoptosis, and several other cellular functions, ranging from metabolism to immune and inflammatory response [3,4,5,6]. In fact, the concentration of GSH and its oxidized form, glutathione disulfide (GSSG), mainly determines the redox state of the cell. Together with other thiol redox couples, such as Trx-SH/Trx-SS, GSH/GSSG acts as a sensor of intracellular redox changes and couples these modifications to redox-dependent biochemical processes [7]. What are the mechanisms by which GSH/GSSG plays a role in signaling? Keeping in mind that the GSH/GSSG couple is the main determinant of H_2_O_2_ level, which is a well-known intracellular messenger in signal transduction (see reviews [8,9,10,11,12,13]), it can play a direct role in signaling through glutathionylation, that is, the formation of mixed disulfides between its cysteine and the cysteine of a protein [14]. As for the processes regulated by phosphorylation, the redox-mediated signal impacts proteins through a specific amino acid residue (i.e., cysteine). Several modifications of the thiol group can occur for structural needs, such as inter/intrachain disulfide –SS–, or different degrees of oxidative stress, such as the sulfenic –SOH, sulfinic –SO_2_H, sulfonic –SO_3_H, and mixed disulfide with cysteine or glutathione –SSR [15]. A prerequisite for cysteine residue modification is its accessibility by the solvent and a peculiar reactivity imposed by near amino acids. Indeed, the pK value of the cysteine thiol group is around 8.0 and, therefore, always protonated at physiological pH. The so-called “reactive cysteine”, instead, is localized in a basic milieu that stabilizes the thiolated form –S^−^ and represents a very reactive group. The formation of glutathionylated proteins (GSSPs) has been known for many years. Initially considered an effect of oxidative stress and damage [16,17], it then became clear that it occurred also under physiological conditions and, being a reversible process, could have a role in redox signaling [18,19,20]. GSSPs can be formed by different biochemical reactions: through the formation of sulfenic acids (reaction below (1)) or the thiol–disulfide exchange that occurs between GSSG and protein thiols (2). The reaction can be reversibly catalyzed by different enzymes belonging to the family of thiol–disulfide oxidoreductases or redoxins, including glutaredoxin (Grx) [21], where reversibility and catalysis are important requirements for the regulatory role of the process [22,23].

(1) PSOH + GSH ⇄ GSSP + H_2_O

(2) PSH + GSSG ⇄ GSSP + GSH

redoxins

It is known that several proteins undergo glutathionylation, resulting in different biological effects [23,24]. Most studies on the identification of glutathionylated proteins have focused on cellular proteins, including proteins involved in host response to infections, but there is a growing number of reports showing that microbial proteins also undergo glutathionylation [25].

Therefore, this review is an attempt to highlight the signaling role of GSH through glutathionylation, particularly focusing on microbial GSSPs and host GSSPs involved in the immune/inflammatory response to infection.

## 2. Glutathionylation in Infections

### 2.1. Viral Infections

It is well known that viruses alter the intracellular redox state to pro-oxidant conditions, which is an alteration that contributes to viral pathogenesis [26,27,28,29,30,31]. Thus, researchers have been evaluating redox signaling as a potential target for antiviral strategies [32,33,34,35,36,37,38]. Virus-induced redox imbalance has been measured in terms of ROS overproduction and/or GSH depletion in different viral infections, with different kinetics and mechanisms according to the type of virus and cell host [39,40,41,42,43,44,45,46]. Based on what has been discussed so far—protein glutathionylation is a physiological process amplified by oxidative stress, and viruses induce a redox imbalance towards a pro-oxidant state—it is not surprising to wonder if glutathionylated proteins are detectable during viral infections and if viral proteins themselves undergo glutathionylation.

The first evidence for viral protein glutathionylation was provided by Davis et al. in 1996 [47]. In fact, they showed that HIV protease contains two cysteines, which are highly conserved among clinical isolates, that could form mixed disulfides with low-molecular-weight (LMW) thiols, including GSH, and that this post-translational modification changes the enzyme activity [47]. HIV protease activity could be regulated through the glutathionylation of these two conserved cysteines by human thioltransferase or glutaredoxin, which has been detected in HIV virions [48]. So, the fine regulation of protease activity may promote optimal viral replication [49]. Interestingly, the finding that the protease of another retrovirus, HTLV-1, could be regulated by reversible oxidation in a similar way to that observed for HIV protease led to the idea that this could be an important evolutionarily conserved mechanism that could prevent the premature activation of retroviral proteases in infected cells—detrimental for the cells and the virus—and promote their activation later in the virus life cycle at the optimal timing for viral maturation and budding [50]. More recently, glutathionylation of chikungunya virus nsP2 protein has been described [51]. In particular, both the recombinant protein and the viral protein in chikungunya-infected cells have been shown to undergo glutathionylation. Interestingly, as well as retrovirus proteases, the glutathionylated protein showed lower protease activity, although the six cysteines identified as being glutathionylated are not in the protease active site. This suggested that glutathionylation induces conformational changes in the protein that can also affect sites far from those that have been glutathionylated, and in this way, affect protein function. Moreover, and again in a similar way to that observed in retrovirus, the specific timing in which the authors observed the modification suggests that the glutathionylation/deglutathionylation process switches the protein function for the progression of the virus life cycle. The same authors showed that dengue and Zika NS5 proteins were glutathionylated in three cysteines and that the post-translational modification reduced the guanylyltransferase activity of the enzyme from both viruses. The fact that the cysteines found to be glutathionylated are highly conserved across more flaviviruses also led these authors to suggest that glutathionylation may be a general mechanism for this family of viruses to regulate NS5 activities. However, how this modification alters the multiple other functions of the protein and how this could affect viral replication remain to be elucidated [52]. Finally, we have data (Checconi et al., unpublished) supporting the glutathionylation of influenza virus proteins that led us to strongly share the hypothesis that the glutathionylation/deglutathionylation process could be an important, common mechanism of redox regulation in the replication of more families of viruses.

The cellular redox proteome responds through modifications, in particular of cysteines, to environmental challenges, such as viruses [53]. Glutathionylation of cellular proteins during viral infections has been reported since 1997. Indeed, Ciriolo et al. [42] measured a significant amount of glutathione-mixed disulfides in epithelial cells infected by Sendai, a parainfluenza virus [42]. Ghezzi et al. [54] measured the rate of protein glutathionylation/deglutathionylation in HIV-infected T lymphocytes, showing that HIV infection made the dethiolation process slower, and the effect was reverted by the addition of the antioxidant N-acetylcysteine (NAC) [54]. Subsequent studies identified several of these proteins that undergo glutathionylation during viral infections; interestingly, some of them belong to pathways involved in the cellular response to infection. It has been reported that interferon regulatory factor 3 (IRF3) is deglutathionylated by Grx during Sendai virus infection to efficiently activate the transcription of interferon genes and therefore the innate immune response to the virus [55]. In the same line, it has been shown that HSV infection induced the production of ROS that mediate the glutathionylation of proteins belonging to the TRAF family, in particular, TRAF3 and TRAF6, which are essential for the proper activation of the antiviral innate immune response [56]. Among the functions influenced by the cysteine proteome, there is also protein secretion [57]. Thanks to a redox proteomic technique based on the use of biotinylated glutathione, a set of proteins, including redoxins such as peroxiredoxins (PRDXs) and thioredoxin (TRX), was found by our group to be released in glutathionylated form by lipopolisaccharide (LPS)-stimulated macrophages and influenza-virus-infected cells [31]. We can speculate that these GSSPs could have a role in the pathogenesis of influenza, as PRDX2 has been demonstrated to be a danger signal in an inflammatory setting [58]. Moreover, a redistribution of key antioxidant enzymes, including PRDX6, in lung cell types was already observed during influenza pneumonia in mice [59]. Thioredoxin deserves special attention, as it is a redox enzyme that has long been studied in the context of infection and inflammation [60,61], including those from influenza virus. In particular, Go et al. [62] demonstrated that TRX1 localization in cell nuclei increased the severity of the disease and lethality in influenza-virus-infected mice by activation of redox-sensitive inflammatory transcription factors; on the other hand, the overexpression of TRX or the administration of recombinant TRX, or a serum albumin-TRX fusion protein, in mice has been shown to ameliorate the outcome of influenza virus lung injury [63,64,65]. These apparent opposite results indicate the importance of redox compartmentalization [66] and suggest that reductive stress, as well as oxidative stress, can contribute to disease mechanisms due to the disruption of redox signaling and control, more so than the increase in reducing or oxidizing species themselves.

Finally, it must be borne in mind that a significant proportion of the “immunopeptidome”, that is, the peptides stimulating immune cells, have been shown to contain glutathionylated cysteines, with consequences for antigen presentation/T-cell recognition. It has been reported that glutathionylation of a coronavirus peptide reduced CD8 T-cell recognition, so this could be a redox mechanism for the virus to negatively impact the immune response [67].

### 2.2. Bacteria

Growing evidence highlights the importance of LMW thiols and GSH in bacteria [25,68]. GSH is present in Gram-negative and some Gram-positive bacteria (i.e., *Listeria monocytogenes* and *Streptococcus agalactiae*), while other Gram-positive bacteria produce LMW thiols, such as bacillithiol (BSH) and mycothiol (MSH), with similar functions. These functions, as in eukaryotic cells, include detoxification of different reactive species to maintain the redox balance of the bacteria cytoplasm. Recently, however, the direct role of GSH in virulence has been described for different bacteria. For example, it has been shown that GSH activates virulence gene expression in the intracellular pathogen *L. monocytogenes* [69]. Similarly, BSH has been shown to be involved in the virulence of some clinical isolates of methicillin-resistant *Staphylococcus aureus* [70,71], and MSH is essential for the growth and survival of *Mycobacterium tuberculosis* during infection [72].

Thanks to advances in proteomic studies, a growing number of bacteria proteins have been identified to be S-glutathionylated, S-bacillithiolated, or S-mycothiolated, making it plausible that S-thiolation could be the mechanism of redox regulation for these proteins. In *Salmonella typhimurium*, a top-down proteomic approach identified over 500 proteins with 1665 post-translational modifications, among which 25 were s-thiolation. Interestingly, nine s-glutathionylated proteins switched to s-cysteinylation under infection-like conditions, suggesting that this shift could be a novel redox mechanism of infection control [73]. Portman et al. [74] demonstrated that the cytolysin listeriolysin O (LLO), a pore-forming virulence factor of *L. monocytogenes*, is reversibly inhibited by glutathionylation. Interestingly, a mutant of LLO unable to undergo this post-translational modification was less virulent in vivo, suggesting that the modification could be important to restrict the pore-forming capability to an appropriate temporal and/or spatial compartment during infection.

Regarding cellular proteins involved in the response to bacterial infection, the death receptor Fas, known to be activated by several pathogens, including *Pseudomonas aeruginosa*, has been shown to be glutathionylated after caspase-dependent cleavage of Grx1. Glutathionylation of Fas then resulted in an amplification of apoptosis [75]. In a subsequent study, the same authors demonstrated Fas glutathionylation in patients with *P. aeruginosa* pneumonia, showing that the modification promoted bacteria clearance. Overall, these results indicate the importance of the Grx1/Fas glutathionylation axis in the resolution of the disease [76]. Recently, it was found that GSH metabolism affects host cytokine production in response to *Borrelia burgdorferi* infection, possibly through glutathionylation. Moreover, GSH-related metabolites have been found to be altered in patients with early onset of Lyme disease and these alterations persist for months after primary infection. Thus, it has been suggested that GSH metabolism and glutathionylation could be important processes in the pathogenesis of the *B. burgdorferi*-induced Lyme disease and, potentially, other inflammatory diseases as well [77].

## 3. Glutathionylation in Inflammation

The final section of this review is dedicated to a brief analysis of glutathionylation in inflammation, focusing on nuclear factor kappa β transcription factor (NFκB)’s glutathionylation.

NFκB is a transcription factor that plays an important role in the activation of proinflammatory response. Under physiological conditions, NFκB is sequestered in the cytoplasm in a latent form by inhibitor κB (IκB). After phosphorylation by IκB kinase (IKK), IκB undergoes degradation via proteasomes and NFκB is then free to move into the nucleus, where it induces transcription of proinflammatory genes. Various studies have shown that glutathionylation, through the bond of GSH to the DNA binding domain, is able to regulate the activity of many transcription factors, including NFκB [78,79]. In particular, the p65 subunit of NFκB has been reported to be glutathionylated in different conditions, such as after supplementation of GSH, which leads to DNA binding inhibition [79]. It has been also demonstrated that the treatment of endothelial cells with an electrophilic compound (cinnamaldehyde) induced p65-glutathionylation, leading to the inhibition of TNF-α-induced p65 nuclear translocation and ICAM-1 expression [80]. During treatment with cinnamaldehyde, the authors also observed an increase of Grx1 protein level. The downregulation of Grx1 abolished p65-glutathionylation and blocked the inhibition of its nuclear translocation and ICAM-1 expression, suggesting that the enzyme is involved in cinnamaldehyde-mediated NFκB inhibition. In a subsequent study, it was shown that treatment with another electrophilic compound, 15-deoxy-Δ12,14-prostaglandin J (2) (15d-PGJ (2)), caused p65-glutathionylation, again suppressing p65 nuclear translocation and the expression of ICAM-1, thus working as an anti-inflammatory molecule [81]. Treatment of hepatocytes with H_2_O_2_ also induced p65-glutathionylation and its retention in the cytoplasm, leading to an increase of the apoptotic rate of hepatocytes [82]. Besides p65, S-glutathionylation was also observed for the p50 subunit of NFκB on Cys-62. This post-translational modification reversibly inhibited its DNA binding activity [83].

Moreover, IKK, the protein responsible for IκB phosphorylation and NFκB translocation to the nucleus, can be inactivated by S-glutathionylation of Cys-179 [84]. So, the NFκB complex, as well as upstream proteins such as TRAF6, is negatively regulated via glutathionylation [85]. In these two studies, it was shown that knockdown of Grx1 hampered IKK and TRAF6 deglutathionylation, respectively, and reduced NFκB activation, indicating the importance of Grx1-mediated deglutathionylation in modulating the NFκB signaling pathway [85]. Moreover, both p65 and IKKa have been shown to be regulated by the Grx1/glutathionylation axis in IL-17A-stimulated epithelial cells, contributing to IL-17A-induced proinflammatory response [86].

Jones et al. [87] demonstrated that glutathione S-transferase (GSTP) is another enzyme capable of inducing the S-glutathionylation of many proteins under conditions of oxidative stress. The authors highlighted that, in unstimulated cells, GSTP bound the NFκB inhibitor, IκBα, while exposure to LPS led to their dissociation. LPS treatment also induced GSTP association with IKKβ and IKKβ-S-glutathionylation to increase. The inhibition of GSTP with siRNA, causing a decrease in IKKβ-S-glutathionylation, enhanced NFκB nuclear translocation, its transcriptional activity, and production of proinflammatory cytokines [87].

Finally, we have to mention that interleukin-1β, together with a group of cytokines, has been shown to undergo glutathionylation itself [88]. In particular, the authors demonstrated that IL-1β glutathionylation at the conserved cysteine Cys-188 protected it from ROS deactivation, making it necessary for maintaining cytokine bioactivity. Grx1 is the physiological negative regulator of the glutathionylation process, and it has been suggested to be a new potential therapeutic target in the treatment of various infectious and inflammatory diseases.

**Figure 1:**
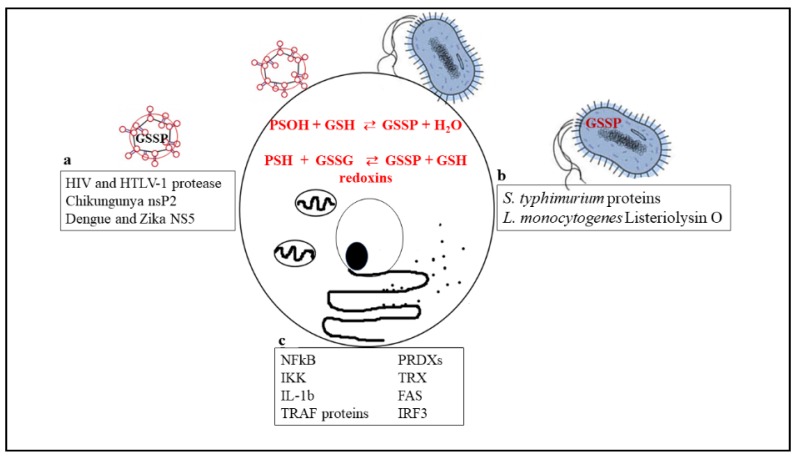
Biochemical reactions through which glutathionylated proteins can be formed and a list of proteins that have been shown to undergo glutathionylation in viruses (**a**), bacteria (**b**), or cellular immune/inflammatory pathways (**c**).

**Figure 2:**
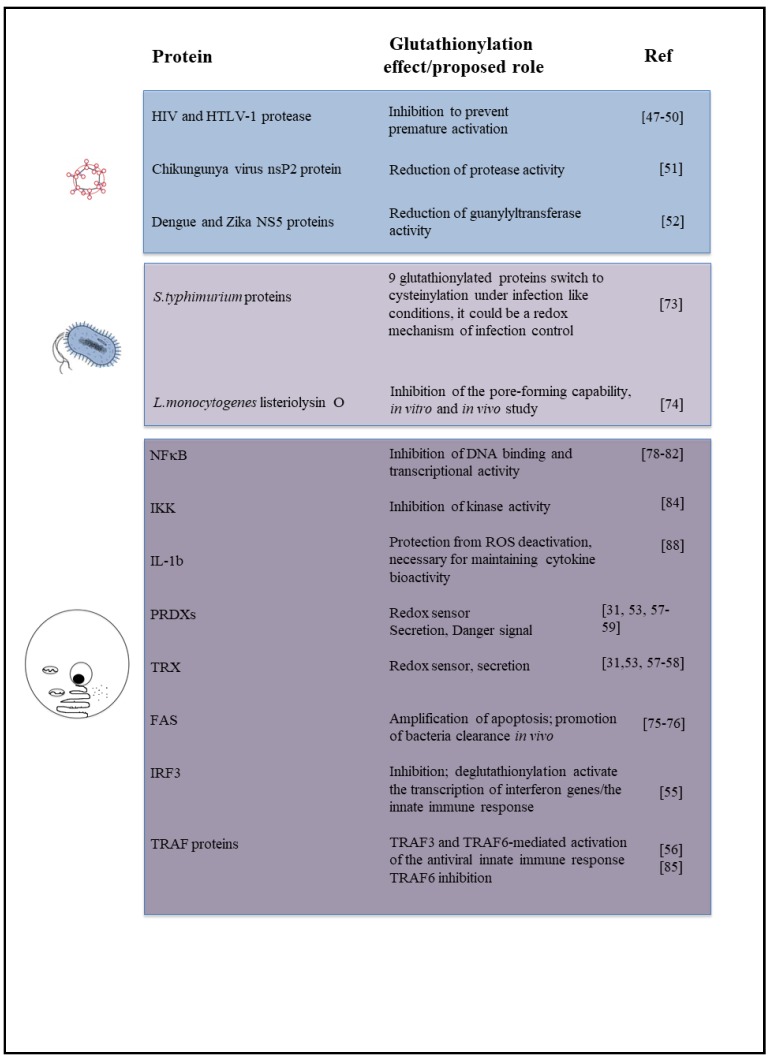
Diagram summarizing the effect or biological role, known or proposed, of glutathionylation on proteins described in this review, including the relevant references.

Figure 1 is a schematic picture of the glutathionylated proteins described in this review, and Figure 2 summarizes the effects of glutathionylation on those proteins.

## 4. Conclusions

In this review, we underscored the role of glutathionylation in viral/bacterial infections and host response. It is well known that infections, particularly viral infections, cause a redox imbalance towards pro-oxidant conditions in the cell, and we believe that glutathionylation fits into these virus-induced changes to regulate its replication and/or cell response. However, based on the available knowledge, we may speculate that the glutathionylation/deglutathionylation process is not a mere consequence of the oxidative burst but a finer regulation of the different phases of viral replication. In particular, glutathionylation could inhibit the premature activation of some viral protein functions, as in the case of retroviral proteases, which would be harmful for the cells and the virus and promote their activation at the optimal timing for viral maturation and spread. In a similar way, the process could play a role in bacterial infections, representing a potential mechanism of virulence. At the same time, the inflammatory cell response to infections could be affected, for example, by the redox processes regarding NFκB activation. This nuclear transcription factor was among the first identified to be redox modulated and strictly involved in inflammation, viral replication, and cell proliferation/apoptosis. The transient glutathionylation of NFκB could also take part in the regulatory processes committed by infections.

Overall, a deeper knowledge of the chemical and regulatory processes through which glutathionylation occurs could pave the way for the identification of novel targets to control microbial infections and related host responses.
